# Biological characteristics of the rtA181T/sW172* mutant strain of Hepatitis B virus in animal model

**DOI:** 10.1186/1743-422X-9-280

**Published:** 2012-11-21

**Authors:** Jie Dai, En-Qiang Chen, Lang Bai, Dao-Yin Gong, Qiao-Ling Zhou, Xing Cheng, Fei-Jun Huang, Hong Tang

**Affiliations:** 1 Center of Infectious Diseases, West China Hospital, Sichuan University, Chengdu, 610041, China; 2 Division of Infectious Diseases, State Key Laboratory of Biotherapy, Sichuan University, Chengdu, 610041, China; 3 Department of Pathology, West China Second Hospital, Sichuan University, Chengdu, Sichuan, 610041, China; 4 Department of Forensic Pathology, Medical School of Basic and Forensic Sciences, Sichuan University, Chengdu, Sichuan, 610041, China

**Keywords:** Hepatitis B virus, rtA181T/sW172* mutation, Transcription and replication, Hepatitis B surface antigen, Secretion defect, Drug sensitivity

## Abstract

**Background:**

The effects of Hepatitis B virus (HBV) rtA181T/sW172* mutation on viral replication and pathogenicity was concerned recently. This study aimed to investigate the biological characteristics of rtA181T/sW172* mutant strain of HBV in animal model.

**Methods:**

The rtA181T/sW172* mutant plasmid was constructed using the pHBV4.1 (wild type HBV) as a template. The wild and mutant HBV replication mouse models were established utilizing a hydrodynamic technique. The titers of hepatitis B surface antigen (HBsAg), hepatitis B e antigen, and HBV DNA in serum, and the levels of HBsAg, hepatitis B core antigen(HBcAg), HBV DNA replication intermediates (HBV DNA RI) and HBV RNA in liver were measured after 1, 3, 5, 7, 10, 12 and 15 days of plasmid injection.

**Results:**

In wild-type HBV replication mouse model, serum HBsAg was high on day 1, 3, and 5, but became lower since day 7; while in mutant HBV mouse model, serum HBsAg was always at very low level. In liver tissues, HBV DNA RI of wild type HBV was detected on day 1 after transfection. The level subsequently peaked on day 3, gradually declined after day 5, and was almost undetectable on day 10. However, the HBV DNA RI levels of the mutant strain were always higher and lasted longer until day 15. Consistently, the expression levels of HBsAg and HBcAg in liver of the mutant group were significantly increased.

**Conclusions:**

In the case of the HBV rtA181T/sW172* mutation, the secretion of serum HBsAg was impaired, whereas HBV DNA replication and HBsAg/HBcAg expression were increased in liver. These results suggest that the mutation can impair HBsAg secretion, and may cause the accumulation of viral core particles in liver.

## Background

More than 350 million people are chronically infected with HBV worldwide, which leads to about 1 million death per year [1]. There are currently two classes of antiviral agents for chronic hepatitis B (CHB): nucleos(t)ide analogs (NAs) directly inhibiting HBV DNA replication and interferon-based therapies that may modulate the host immune response as well as viral replication. However, widespread use of NAs in the clinics might have contributed to the increased incidence of drug resistant cases.

HBV, a member of the hepadnavirus family, is an enveloped virus that contains a partially-double stranded circular DNA about 3.2 kb in length. HBV DNA has a very compact coding organization with four partially overlapping open reading frames (ORFs), including ORF P, X, S and C [2]. Among them, ORF P that encodes the RT (reverse transcriptase) domains of the polymerase overlaps completely with the ORF S that encodes HBV surface proteins [3]. The HBV surface proteins, including the S protein (226 amino acids), the M protein (281 amino acids), and the L protein (389–400 amino acids), are encoded by one long open reading frame that utilizes three different start codons and the same stop codon. The major functions of the HBV surface proteins include envelopment of nucleocapsid with subsequent assembly of virion, and assembly into empty subviral particles that are secreted in great excess over HBV virions collectively referred to as hepatitis B surface antigen [4]. The surface proteins also are targets of the host cellular immune system. The extent to which the host recognizes viral antigens presented by infected cells determines whether an infection will be acute or chronic.

The HBV rtA181T/sW172* mutant strain researched in this study is that the rtA181T mutation in the viral polymerase encodes a stop codon in the overlapping surface gene at amino acid 172 (sW172) resulting in truncation of the last 55 amino acids of the C-terminal hydrophobic region of the surface proteins. The rtA181T mutation causes drug resistance to adefovir (ADV) clinically [5]. It has been revealed in cell culture that the mutation results in a decreased susceptibility to lamivudine (LAM), ADV, and tenofovir, while remaining sensitive to entecavir (ETV) [6]. The rtA181T/sW172* mutation also reduces the typical extent of virological breakthrough [4]. Previous studies in vitro also demonstrated that the rtA181T/sW172* mutation may impair HBsAg secretion, and may be an oncogenic potential factor leading to advanced hepatocellular carcinoma (HCC) [7]. However, the effect of the rtA181T/sW172* mutation on HBV virology in vivo remains unclear. So it is thus necessary to study the biological characteristics of the HBV rtA181T/sW172* mutation in vivo environment.

In this study, a mouse model for the replication of the HBV rtA181T/sW172* mutant was established using a hydrodynamic-based procedure [8]. The effect of the rtA181T/sW172* mutation on HBV transcription, replication and HBsAg secretion were investigated.

## Results

### Expression levels of HBsAg and HBeAg in mouse serum

**Figure 1:**
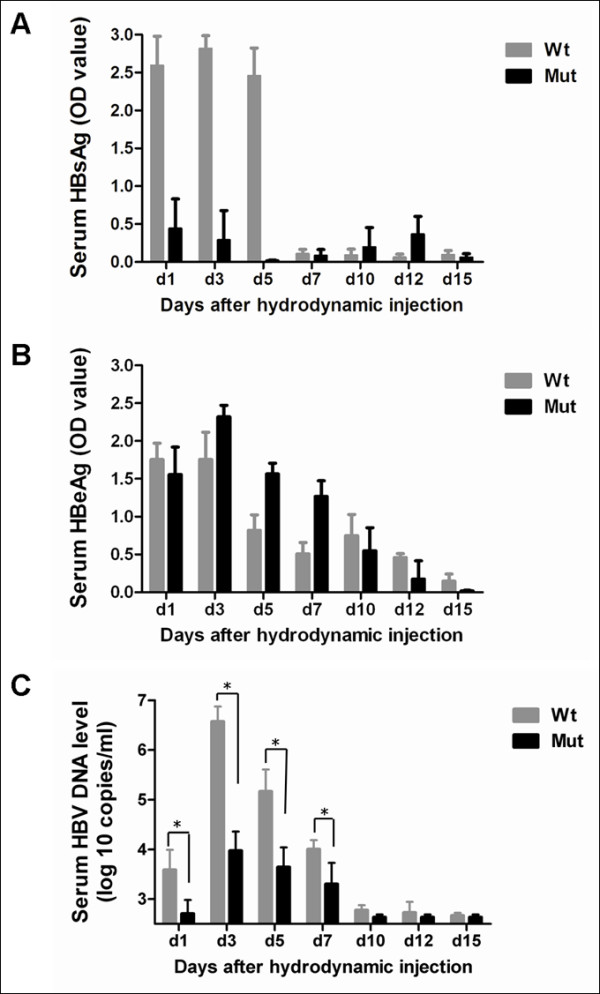
**Time-dependent HBV antigen and HBV DNA expression in mice serum after hydrodynamic transfection in vivo.** Mice were injected with 10μg pHBV4.1 (Wt) or pHBV rtA181T/sW172* (Mut) respectively. At different time points, HBsAg (**A**) and HBeAg (**B**) in the serum were measured by ELISA, and HBV DNA (**C**) in the serum were detected by quantitative real-time PCR. The mean HBsAg, HBeAg and DNA levels plus standard deviation (indicated by error bars) from three independent analyses are shown (* P<0.001).

After injection of the wild type (pHBV4.1) or the mutant (pHBVrtA181T/sW172*) plamids, the level of serum HBsAg in mice injected with the wild type plasmid was very high (OD>2.4) on day 1, 3, and 5, and became low (OD<0.2) since day 7. However, the level of serum HBsAg in mice injected with the mutant plasmid was always very low (OD<0.4, Figure 1A). In contrast, serum HBeAg had similar patterns between two groups (Figure 1B).

### HBV-DNA level in mouse serum

Compared to wild type, the rtA181T/sW172* mutant strain showed an approximately 0.8 log reduction on day 1, 2.6 log reduction on day 3, 1.5 log reduction on day 5, and 0.7 log reduction on day 7 in serum HBV DNA titers. The serum HBV DNA levels of the mutant strain were always lower than that of wild type. The difference was significant on day 1, 3, 5 and 7, respectively (Figure 1C). Since day 10, viral loads decreased to very low level for both two groups.

### HBV RNA transcription level in mouse liver

**Figure 2:**
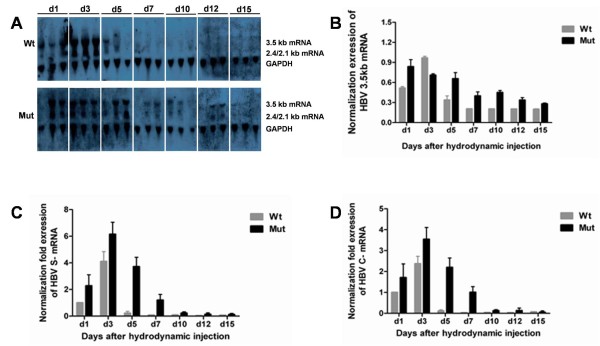
**Time-dependent viral transcription after hydrodynamic transfection in vivo.A**: HBV RNA of mice liver were detected by Northern blotting, and GAPDH transcript was used as an internal control for RNA loading per lane; **B**: The 3.5-kb HBV mRNA was quantitatively calculated automatically by the Quantity-One software (Bio-Rad); **C** or **D**: The total levels of HBV S-mRNA or C-mRNA in mouse liver were detected by qPCR, and the mean value of Wt of day 1 was defined as 1.

In order to investigate the transcriptional character of the HBV rtA181T/sW172* mutant strain, HBV RNA levels in mouse liver were evaluated by northern blotting analysis (Figure 2A, B) and qPCR (Figure 2C, D). For mice injected with the wild type HBV plasmid, the level of the 3.5kb HBV mRNA peaked on day 3, significant reduced on day 5, and became almost undetectable on day 7 (Figure 2A). But for mice injected with the mutant HBV plasmid, the 3.5kb HBV mRNA was detected on day 1, reached peak on day 3–5, decreased on day 7, and still remained detectable until day 15 (Figure 2A). At most of the time points, the 3.5kb HBV mRNA level in mice injected with the mutant HBV plasmid was higher than that of mice injected with the wild type plasmid (Figure 2B). Meanwhile, compared to mice received injections of the wild type plasmid, the levels of HBV S-mRNA and C-mRNA of mice injected with the mutant plasmid were increased as detected by qPCR (Figure 2C, D). The values shown were the average of three experiments (means plus standard deviation).

### Levels of HBV DNA replication intermediates in mouse liver

**Figure 3:**
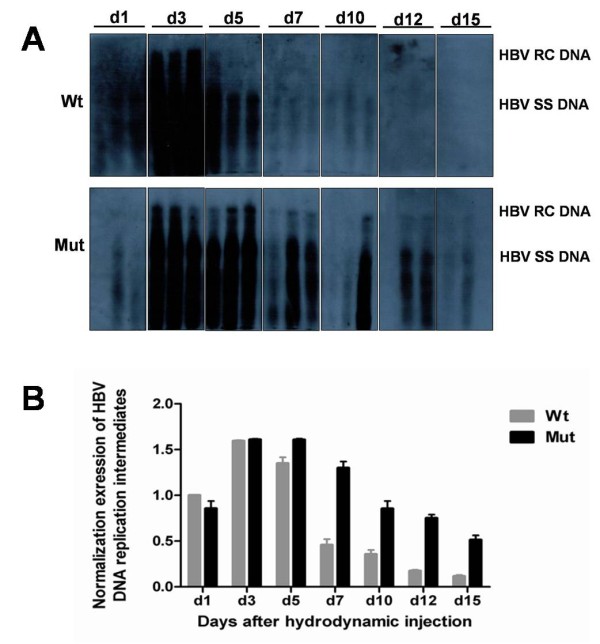
**Time-dependent viral replication after hydrodynamic transfection in vivo.** Mice were injected hydrodynamically with 10μg pHBV4.1 (Wt) or pHBV rt A181T/sW172 (Mut) respectively, and were sacrificed at different time points. **A**: HBV DNA replication intermediates of mice livers were detected by Southern blotting. **B**: Quantitative analysis of HBV DNA replication intermediates. The levels of HBV DNA replication intermediates in Wt group on day 1 were defined as 1.

In the mouse model for wild type HBV replication, HBV DNA replication intermediates were detectable on day 1, became abundant on day 3, decreased on day 5, and were very weak on day 7 (Figure 3A). However, the HBV DNA replication intermediates levels of the HBV mutant rtA181T/sW172* were detectable from day 1 to day 15 and peaked on day 3 and day 5 (Figure 3B). Compared to the wild type HBV, the HBV DNA replication intermediates levels of mutant HBV in mouse liver were higher and also lasted longer (Figure 3A, B).

### Expression levels of HBsAg and HBcAg in mouse liver

**Figure 4:**
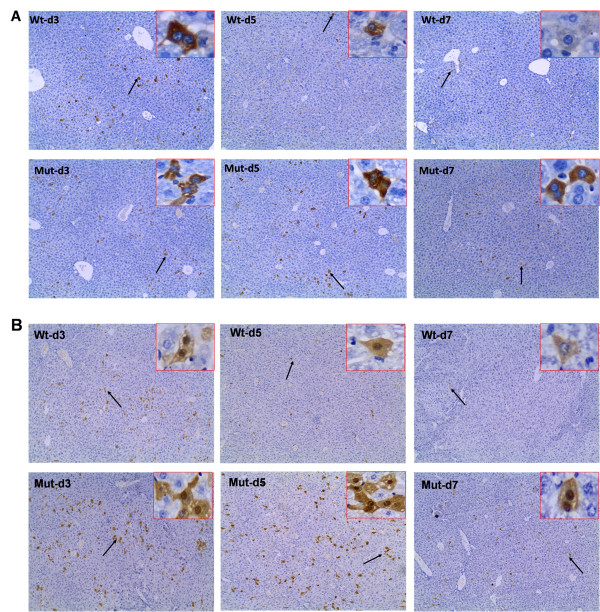
**The expression of HBsAg (A) and HBcAg (B) immunohistochemistry detection in liver tissue sections.** Mice liver sections on day 3, 5 and 7 after transfection were detected using specific antibody. The positive expressions were stained brown as shown by black arrow, which were magnified 400 times in the top right corner of these pictures (100×original magnification).

Animals were sacrificed and livers were embedded and sectioned at different time points after injection. As shown in Figure 4, HBsAg staining can only be detected in the cytoplasm (4A), while HBcAg was present in the nuclei and cytoplasm of the hepatocytes with most expression found in the nuclei (4B). And all the positive cells were stained brown. HBsAg-positive cells in mice infected with wild type HBV were abundant on day 3. The number of positive cells significantly reduced on day 5, and positive cells became scarce in liver on day 7. In contrast, HBsAg stained hepatocytes in sections from the mutant group accumulated in abundance from day 3 to day5, and diminished on day 7 (4A). Meanwhile, the expression pattern of HBcAg was similar to that of HBsAg (4B). It also revealed that the expression level of HBsAg and HBcAg in the livers of the mutant group was increased compared with wild type.

### Inhibition effects of antiviral drugs on wild type and mutant HBV in vivo

**Figure 5:**
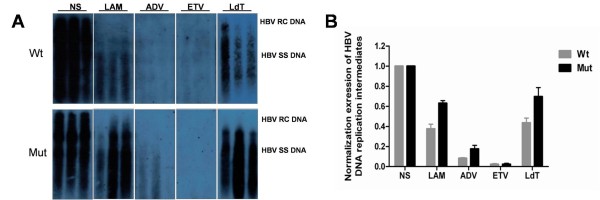
**Drug susceptibility test of antiviral drugs on HBV replication using the HBV replication mouse model.A**: HBV DNA replication intermediates were detected by southern blotting in the mouse model treated with NS (control), lamivudine (LAM), adefovir (ADV), entecavir (ETV) and telbivudine (LdT), respectively. **B**: The relative levels of HBV DNA replication intermediates in the mouse model among different groups. The levels of HBV DNA replication intermediates in NS treated wild-type group and mutant mice were set to 1 respectively, and the relative levels of treated groups were calculated accordingly.

To analyze the effects of the HBV rtA181T/sW172* mutation on antiviral drug resistance, the inhibitory effects of four anti-HBV drugs on replication of the wild type and mutant HBV were compared. For wild type HBV, viral replication was inhibited by LAM, ADV, ETV and telbivudine (LdT) for 6.19-fold, 9.57-fold, 9.82-fold and 5.01-fold, respectively (Figure 5A, B). For the mutant HBV, viral replication was inhibited by LAM, ADV, ETV and LdT for 3.83-fold, 7.80-fold, 9.80-fold and 3.27-fold, respectively (Figure 5A, B). Compared to wild type HBV, the rtA181T mutant remained sensitive to ETV, but had a reduced susceptibility to LAM, ADV and LdT, as the inhibition effects decreased about 2.36-fold, 1.77-fold and 1.74-fold (Figure 5).

## Materials and methods

### Ethics statement

This study obtained ethics approval from the Laboratory Animal ethics committee of Sichuan University.

### Plasmid

The wild type pHBV4.1 (pHBVwt [wt, wild-type]) is an HBV replication competent plasmid which contains 1.3 copies of the HBV genome (subtype ayw) [9] and is capable of HBV transcription, replication, and expression both in vitro and in vivo [10]. A point mutation (rtA181T) in the RT region replacing Alanine (A) 181 with Threonine (T) was introduced via site-directed mutagenesis (QuikChange mutagenesis kit, Stratagene) according to the manufacturer’s instructions using the forward primer: 5'-CAGCCCGTTTCTCCTGACTCAGTTTACTAGTGC-3' and the reverse primer: 5'-GCACTAGTAAACTGAGTCAGGAGAAACGGGCTG-3'. The resulting mutant plasmid was named pHBVmut (mut, mutation type). This mutation also created a premature stop codon that replaces amino acid 172W of HBsAg.

### A mouse model for HBV replication

Male BALB/C mice that were maintained in specific-pathogen-free (SPF) conditions and weighted 18-20g were purchased from the Laboratory Animal Center at Sichuan University. All mice received humane care under the Institutional Review Board in accordance with Animal Protection Art of Sichuan University.

In order to establish the mouse model for HBV replication, 10μg of pHBV4.1 or pHBVmut plasmids diluted in phosphate-buffered saline (PBS) was injected into the mouse tail vein in a volume of 10% of the body weight (v/w) within 5–8 seconds (hydrodynamic in vivo transfection) [11]. Mice were sacrificed after 1, 3, 5, 7, 10, 12 and 15 days of DNA injection. Liver tissues and blood were collected.

### HBV antigen assays

Serum HBsAg and HBeAg were measured using ELISA kits following the manufacturer’s protocol (Shanghai Shiye Kehua Company, China). Absorbance was measured in a microtiter plate reader with dual-wave length measurement (450/645nm).

Formalin-fixed liver tissues from the treated mice were embedded in paraffin. HBsAg and HBcAg were detected by immunohistochemical staining using specific antibodies against HBsAg (mouse anti-HBs, Thermol) and HBcAg (rabbit anti-HBc, NEOMARKERS), respectively. All procedures for the immunological detections of these antigens were performed according to the manufacturer's protocol.

### Analysis of liver HBV RNA

Mouse liver tissues were mechanically pulverized in liquid nitrogen, and total RNA was extracted from pulverized tissues using Trizol reagent following the manufacturer’s instructions (Invitrogen, USA). The 30 μg HBV RNA was analyzed by Northern blotting hybridization using probes for both GAPDH and HBV, with the GAPDH serving as an internal control [12].

### Analysis of liver HBV DNA replication intermediates

Frozen liver tissues were mechanically pulverized in liquid nitrogen and HBV DNA replication intermediates were isolated from one hundred and twenty micrograms of liver tissue powder as described previously [12]. And these HBV DNA replication intermediates were diluted to 30 μl with TE buffer. All of viral replication intermediates was analyzed by Southern blotting as previously described [8]. Membranes were hybridized with digoxigenin-labeled (Roche Applied Science) HBV ayw genomic DNA to detect HBV sequences [13]. The levels of HBV DNA replication intermediates were calculated by the Quantity One software according to manufacturer’s instructions (Bio-Rad).

### Detection of HBV-DNA and HBV-RNA by quantitative real-time PCR

100 μL of mice serum predigested with DNaseIwas used for the detection of HBV DNA by quantitative real-time PCR (qPCR) using a diagnostic kit for quantification of HBV-DNA (Da An Gene, Guangzhou, China).

The qPCR analysis of HBV RNA in mice liver was performed using the LightCycler system (Bio-Rad). Amplification was carried out in two steps: denaturation at 95°C for 5 sec and annealing at 60°C for 5 sec. Also the number of PCR cycles was 30 times. To distinguish specific from nonspecific cDNA products, a melting curve was obtained at the end of each run. Data were normalized against the GAPDH level in each sample. HBV RNA was detected using the C region primer pairs (forward: 5'-CTGGGTGGGTGTTAATTTGG-3', reverse: 5'-TAAGCTGGAGGAGTGCGA AT-3') and the S region primer pairs (forward: 5'-CTCCAATCACTCACCAACCT-3', reverse: 5'-TCCAGA AGA ACCAACAAG AAG A-3'). The primers for GAPDH (internal control) were: forward: 5'-AACTTTGGCATTGTGGAAGG-3', reverse: 5'-ACACATTGGGGGTAGGAACA-3'. (All primers were synthesized by Invitrogen, Shanghai, China).

### Antiviral treatment utilizing NAs

Twenty-four hours after hydrodynamic injection, nucleoside analogues, including lamivudine (GlaxoSmithKline, 250mg/kg/d), adefovir (GlaxoSmithKline, 15mg/kg/d), entecavir (Bristol-Myers Squibb, 0.075mg/kg/d), and telbivudine (LdT, NOVARTIS, 1800mg/kg/d), and normal saline (NS) were administrated via oral gavage to mice injected with either the wild type or the mutant HBV for three times at 24-h intervals. The drug dose was determined based on the human body dosage and experimental experience. Mice were sacrificed 4–6 h after the final administration via oral gavage. Liver tissues were frozen in liquid nitrogen and stored at −70°C for DNA extraction and analysis.

## Discussion

CHB is one of the most common human infectious diseases worldwide. Infected individuals are at a high risk of developing liver cirrhosis and HCC [14]. Treatment of CHB is aimed at suppressing HBV replication, at reducing the accompanying histological inflammation, and at decreasing the risk of cirrhosis and HCC [15]. Therefore, antiviral therapies with NAs or interferon are currently the key treatments for CHB.

However, drug resistance is the major challenge associated with long-term therapy with NAs. Drug resistant HBV mutant strains will gradually become dominant, which leads to disease deterioration and other serious consequences. Many studies showed that treatment of CHB patients with LAM, ADV, or LdT could result in the emergence of the HBV rtA181T mutant [5,16-18]. Clinically rtA181T is a common resistance mutation to ADV. This mutation not only induces a decreased susceptibility to ADV, LAM, and tenofovir [6], but also encodes S gene mutation. It is reported that the nonsence mutation at position 172 in the S gene leads to a 55 amino acids truncation of HBsAg. This mutation is therefore also called HBV rtA181T/sW172*, which may affect the biological characteristics and pathogenesis of HBV [4]. However, the biological features of this HBV mutant in vivo still remain unclear.

Most of recent studies on HBV mutant were done in cultured cells. Transgenic mice [19,20] are useful models to study HBV. However, establishing specific transgenic mice is time and cost consuming. The HBV replication mouse model that we established with a hydrodynamic-based procedure is a rapid and convenient alternative, especially for the characterization of HBV mutations. We have constructed the rtA181T/sW172* mutant strain of HBV and investigated its biological characteristics in vivo with our HBV replication mouse model.

Firstly, we found that serum HBsAg level of the rtA181T/sW172* mutant was very low (OD<0.4) from day 1 to day 15 after in vivo transfection (Figure 1A), and that the serum HBV DNA level of the mutant was lower than that of wild type (Figure 1C). This may be explained by the fact that the rtA181T/sW172* mutation causing the production of truncated HBsAg, which cannot be secreted into the serum. HBsAg plays an important role in the packaging and secretion of virion [21]. The complete HBV virion consists of an icosahedral nucleocapsid core and an outer lipid envelope, in which the three envelope proteins [small (S), medium (M) and large (L)] are anchored as transmembrane proteins playing a major role in HBV morphogenesis and infectivity. And the mature nucleocapsid enveloped by the surface proteins can be released to serum [22]. As the S gene overlaps the P gene completely, the A→T mutation at position 181 of the P gene in the RT domain replaces the amino acid tryptophan 172 of the S gene with a termination codon, resulting in the truncation of the last 55 amino acids. All of the HBV surface proteins (S protein, M protein, L protein) decrease approximately 6 kDa in molecular weight. These truncated surface proteins may affect the virus assembly and secretion. In this study, the mostly HBV virions and HBsAg assembled by these truncated surface proteins may not be secreted into serum and thus became accumulated in liver. It seems that the function of the truncated 55 amino acids in the C-terminal is critical for HBV assembly. The rtA181T/sW172* mutation thus leads to defects in the assembly and secretion of HBsAg and virion, which results in decreased levels of serum HBsAg and HBV DNA.

Detection of HBsAg is achieved by antibody-based assays targeting the ‘a’ determinant, the highly homologous region within HBsAg, which is also used as the main target by hepatitis B vaccines [23]. Recognition of the ‘a’ determinant by antibody against HBsAg (anti-HBs) depends on its 3D conformation, which also relies on the amino acid sequence of the regions flanking the ‘a’ determinant [24]. However, mutants of HBsAg continue to evolve as a result of vaccine escape, immune selection, and an error prone reverse transcriptase [25]. Because the sW172* mutation of HBsAg gene is not inside the ‘a’ determinant, currently commercially antibody for truncated HBsAg is effective. In addition, the immunohistochemistry results of HBsAg in liver tissue also supported the effectiveness of conventional antibody for truncated HBsAg detection. So the low serum HBsAg in mutant HBV mice model should be due to the intracelluar retention of truncated HBsAg. As detection of HBV DNA in serum is qPCR-based assays, the HBV DNA can be observed even if it is very little. Compared with the wild mice, the level of HBV DNA in mutant mice serum decreased significantly, and these finds were consistent with the conditions of patients who had the HBV rtA181T/sW172* mutation.

However, the synthesis and secretion of HBeAg, a secretory antigen, is regulated differently from HBsAg, and is regulated by HBV C open reading frame. Serum HBeAg of two groups showed similar patterns.

Secondly, our study revealed that the expression levels of HBsAg and HBcAg in liver of mice infected with the mutant HBV were higher than those of wild type, especially on day 5 and day 7 (Figure 3). HBsAg, the envelope protein, is normally secreted into serum following infection. HBcAg, the nucleocapsid protein, is synthesized in infected cells and is required for HBV viral replication. It seems that the mutant HBV has defects in the secretion of virions and HBsAg, leading to the accumulation of a large number of viral particles and HBsAg in liver.

The expression levels of HBV RNA and DNA of the mutant HBV were higher and lasted longer in liver than wild type (Figure 2, 3), which may be due to the increased capability of mutant HBV transcription and replication . To classify this issue, it is necessary to study the promoter activity of the mutant HBV in the future research. As compared to wild type HBV strain, the transcription and replication levels of the HBV rtA181T/sW172* mutant strain in vivo delayed in reaching the peak level and lasted significantly longer, and the mutation also was demonstrated HBsAg and HBcAg continued retention in liver. And these antigens in the liver tissue may stimulate prolonged immune response. Previous evidences showed that the adaptive immune response should be responsible for viral clearance and disease pathogenesis during HBV infection [26]. Though HBV is not directly hepatotoxic [27,28], recent studies of HBV insertions in HBV-related HCCs revealed that HBV integration can target the telomerase reverse transcriptase gene, suggesting a potential role for viral insertion in HBV-related carcinogenesis [29-31]. The cytotoxic T lymphocyte (CTL) not only kills the virus, but also kills the infected hepatocyte, leading to liver damage. Persistent infection of HBV is characterized by chronic liver cell injury, regeneration, inflammation, widespread DNA damage, cirrhosis of the liver, and hepatocellular carcinoma [32]. Further studies are ongoing to investigate where and why the secretion of the mutant HBsAg and HBV virions is impaired, and to explore the mechanism of mutant HBV accumulation in liver. The related research will be reported in the future.

Thirdly, cell culture studies have showed that the single amino acid substitution at position rt181 in HBV is the primary resistance mutation to ADV [33] and it also causes cross resistance to LAM and LdT [6]. The concentrations of LAM and LdT for 50% inhibition of HBV replication (IC50) are 0.023μM and 0.335μM, respectively. Our study found that the folds of decrease in the sensitivity of the mutant to LAM, ADV, and LdT are different. However, this study utilized 0.022 μM of LAM, 0.0051 μM of ETV and 0.15 μM of LdT in our mouse models. The corresponding concentration of LAM in human would be 16.67 times the normal oral dose, that of ETV would be equivalent to the normal dose, and that of LdT would have reached 20 times the normal dose. Oral ADV reduced HBV DNA in both serum and liver significantly better than LAM, and also worked well at concentrations as low as 1.0 mg/kg/day [34]. So in this study, the antiviral effect to HBV rtA181T/sW172* is ETV>ADV>LAM>LdT. It is related to the establishment method of the mouse model, individual difference of mice and the drug concentration.

Currently most drug resistance monitoring in clinical practice is achieved by detecting viral load, not by direct sequencing. Serum viral load would not be as high as expected for the kind of variant. This makes us to focus on the HBV rtA181T/sW172* mutation. If the patient carries the variation and we failed to change drug in time, the disease would deteriorate, once rtA181T/sW172* become the dominant strains. However, the mouse model used in this study is a transient HBV infection model. The chronic influence of the mutant virus to mice requires further study.

In conclusion, our results indicated that the rtA181T/sW172* mutation might impair serum HBsAg secretion and reduce serum HBV DNA level. However, the transcription and replication levels of the mutant strain in liver tissues were increased, which may be explained by the accumulation of viral core particles in liver or the enhanced replication ability of this mutant strain. The clinical consequences of infection by these S gene mutants demand further clarification. Judicious selection of antiviral agents and vigilant monitoring of viral mutants during the course of therapy are advised.

## Abbreviations

HBV: Hepatitis B virus; HBsAg: Hepatitis B surface antigen; HBeAg: Hepatitis B e antigen; HBcAg: Hepatitis B core antigen; CHB: Chronic hepatitis B; HBV DNA RI: HBV DNA replication intermediates; qPCR: Quantitative real-time PCR; LAM: Lamivudine; ADV: Adefovir dipivoxil; LdT: Telbivudine; ETV: Entecavir.

## Competing interests

The contents are solely the responsibility of the authors and do not necessarily represent the views of the funding source. The authors declare that they have no competing interests.

## Authors’ contributions

TH conceived the study, provided fund supporting and revised the manuscript critically for important intellectual content. DJ, CEQ, ZQL, BL, GDY, CX and LFJ made substantial contributions to experiment, analysis and interpretation of data. DJ and CEQ participated in interpretation of data and manuscript preparation. All authors have read and approved the final manuscript.

## References

[B1] Lupberger J Hildt E Hepatitis B virus-induced oncogenesis World J Gastroenterol 2007 13 74 81 17206756 10.3748/wjg.v13.i1.74 PMC4065878

[B2] Bartholomeusz A Locarnini S Hepatitis B virus mutations associated with antiviral therapy J Med Virol 2006 78 Suppl 1 S52 S55 16622878 10.1002/jmv.20608

[B3] Michel ML Tiollais P Structure and expression of the hepatitis B virus genome Hepatology 1987 7 61S 63S 10.1002/hep.1840070711 2948897

[B4] Warner N Locarnini S The antiviral drug selected hepatitis B virus rtA181T/sW172* mutant has a dominant negative secretion defect and alters the typical profile of viral rebound Hepatology 2008 48 88 98 10.1002/hep.22295 18537180

[B5] Kim JH Jung YK Joo MK Yim HJ Park JJ Kim JS Bak YT Yeon JE Byun KS Hepatitis B viral surface mutations in patients with adefovir resistant chronic hepatitis B with A181T/V polymerase mutations J Korean Med Sci 2010 25 257 264 10.3346/jkms.2010.25.2.257 20119580 PMC2811294

[B6] Villet S Pichoud C Billioud G Barraud L Durantel S Trepo C Zoulim F Impact of hepatitis B virus rtA181V/T mutants on hepatitis B treatment failure J Hepatol 2008 48 747 755 10.1016/j.jhep.2008.01.027 18331765

[B7] Lai MW Yeh CT The oncogenic potential of hepatitis B virus rtA181T/surface truncation mutant Antivir Ther 2008 13 875 879 19043921

[B8] Liu FJ Liu L He F Wang S Zhou TY Liu C Deng LY Tang H Establishment and primary application of a mouse model with hepatitis B virus replication World J Gastroenterol 2007 13 5324 5330 17879401 10.3748/wjg.v13.i40.5324 PMC4171321

[B9] Tang H McLachlan A Transcriptional regulation of hepatitis B virus by nuclear hormone receptors is a critical determinant of viral tropism Proc Natl Acad Sci USA 2001 98 1841 1846 10.1073/pnas.98.4.1841 11172038 PMC29344

[B10] Tang H McLachlan A A pregenomic RNA sequence adjacent to DR1 and complementary to epsilon influences hepatitis B virus replication efficiency Virology 2002 303 199 210 10.1006/viro.2002.1645 12482672

[B11] Liu F Song Y Liu D Hydrodynamics-based transfection in animals by systemic administration of plasmid DNA Gene Ther 1999 6 1258 1266 10.1038/sj.gt.3300947 10455434

[B12] Tang H Delgermaa L Huang F Oishi N Liu L He F Zhao L Murakami S The transcriptional transactivation function of HBx protein is important for its augmentation role in hepatitis B virus replication J Virol 2005 79 5548 5556 10.1128/JVI.79.9.5548-5556.2005 15827169 PMC1082733

[B13] Gao Z Liu FJ Liu L Zhou TY Lei J Xu L Liu C Dai J Chen EQ Tang H Application of hepatitis B virus replication mouse model World J Gastroenterol 2010 16 1979 1985 10.3748/wjg.v16.i16.1979 20419834 PMC2860074

[B14] Lee WM Hepatitis B virus infection N Engl J Med 1997 337 1733 1745 10.1056/NEJM199712113372406 9392700

[B15] EASL Clinical Practice Guidelines Management of chronic hepatitis B J Hepatol 2009 50 227 242 10.1016/j.jhep.2008.10.001 19054588

[B16] Chien RN Yeh CT Wang PN Kuo MC Hsieh SY Shih LY Liaw YF Acute leukaemia in chronic hepatitis B patients with lamivudine therapy Int J Clin Pract 2004 58 1088 1091 10.1111/j.1742-1241.2004.00266.x 15605678

[B17] Yatsuji H Noguchi C Hiraga N Mori N Tsuge M Imamura M Takahashi S Iwao E Fujimoto Y Ochi H Emergence of a novel lamivudine-resistant hepatitis B virus variant with a substitution outside the YMDD motif Antimicrob Agents Chemother 2006 50 3867 3874 10.1128/AAC.00239-06 16982790 PMC1635170

[B18] Yeh CT Chien RN Chu CM Liaw YF Clearance of the original hepatitis B virus YMDD-motif mutants with emergence of distinct lamivudine-resistant mutants during prolonged lamivudine therapy Hepatology 2000 31 1318 1326 10.1053/jhep.2000.7296 10827158

[B19] Schorr O Borel C Trepo C Zoulim F Hantz O Effects of liver growth factors on hepadnavirus replication in chronically infected duck hepatocytes J Hepatol 2006 44 842 847 10.1016/j.jhep.2005.09.014 16458387

[B20] Schinazi RF Ilan E Black PL Yao X Dagan S Cell-based and animal models for hepatitis B and C viruses Antivir Chem Chemother 1999 10 99 114 10431609 10.1177/095632029901000301

[B21] Lentz TB Loeb DD Roles of the envelope proteins in the amplification of covalently closed circular DNA and completion of synthesis of the plus-strand DNA in hepatitis B virus J Virol 2011 85 11916 11927 10.1128/JVI.05373-11 21900164 PMC3209309

[B22] Bruss V Hepatitis B virus morphogenesis World J Gastroenterol 2007 13 65 73 17206755 10.3748/wjg.v13.i1.65 PMC4065877

[B23] Weber B Diagnostic impact of the genetic variability of the hepatitis B virus surface antigen gene J Med Virol 2006 78 Suppl 1 S59 S65 16622880 10.1002/jmv.20610

[B24] Carman WF Zanetti AR Karayiannis P Waters J Manzillo G Tanzi E Zuckerman AJ Thomas HC Vaccine-induced escape mutant of hepatitis B virus Lancet 1990 336 325 329 10.1016/0140-6736(90)91874-A 1697396

[B25] Lou SC Pearce SK Lukaszewska TX Taylor RE Williams GT Leary TP An improved Abbott ARCHITECT assay for the detection of hepatitis B virus surface antigen (HBsAg) J Clin Virol 2011 51 59 63 10.1016/j.jcv.2011.01.019 21367654

[B26] Chisari FV Isogawa M Wieland SF Pathogenesis of hepatitis B virus infection Pathol Biol (Paris) 2010 58 258 266 10.1016/j.patbio.2009.11.001 20116937 PMC2888709

[B27] Chisari FV Rous-Whipple Award Lecture. Viruses, immunity, and cancer: lessons from hepatitis B Am J Pathol 2000 156 1117 1132 10.1016/S0002-9440(10)64980-2 10751335 PMC1876872

[B28] Guidotti LG Chisari FV Immunobiology and pathogenesis of viral hepatitis Annu Rev Pathol 2006 1 23 61 10.1146/annurev.pathol.1.110304.100230 18039107

[B29] Paterlini-Brechot P Saigo K Murakami Y Chami M Gozuacik D Mugnier C Lagorce D Brechot C Hepatitis B virus-related insertional mutagenesis occurs frequently in human liver cancers and recurrently targets human telomerase gene Oncogene 2003 22 3911 3916 10.1038/sj.onc.1206492 12813464

[B30] Murakami Y Saigo K Takashima H Minami M Okanoue T Brechot C Paterlini-Brechot P Large scaled analysis of hepatitis B virus (HBV) DNA integration in HBV related hepatocellular carcinomas Gut 2005 54 1162 1168 10.1136/gut.2004.054452 16009689 PMC1774867

[B31] Tamori A Yamanishi Y Kawashima S Kanehisa M Enomoto M Tanaka H Kubo S Shiomi S Nishiguchi S Alteration of gene expression in human hepatocellular carcinoma with integrated hepatitis B virus DNA Clin Canc Res 2005 11 5821 5826 10.1158/1078-0432.CCR-04-2055 16115921

[B32] Chisari FV Ferrari C Hepatitis B virus immunopathogenesis Annu Rev Immunol 1995 13 29 60 10.1146/annurev.iy.13.040195.000333 7612225

[B33] Locarnini S Primary resistance, multidrug resistance, and cross-resistance pathways in HBV as a consequence of treatment failure Hepatol Int 2008 2 147 151 10.1007/s12072-008-9048-3 19669299 PMC2716855

[B34] Julander JG Sidwell RW Morrey JD Characterizing antiviral activity of adefovir dipivoxil in transgenic mice expressing hepatitis B virus Antiviral Res 2002 55 27 40 10.1016/S0166-3542(01)00223-6 12076749

